# Hemorrhage as a Sign of Congenital Syphilis

**Published:** 1899-04

**Authors:** 


### Hemorrhage as a Sign of Congenital Syphilis.

In the course of the description of a case of hemorrhagic congenital syphilis appearing as a hemorrhagic vesicular eruption, Dr. William S. Gottheil calls attention to the importance of otherwise unexplainable bleeding in infants as symptoms of congenital lues. They may be the only mark of the disease, especially at first; but they are almost invariably accompanied by a diminution of the coagulability of the blood similar to that of hæmophilia, and the case usually goes on rapidly to a fatal termination. Disease of the vascular walls is one of the commonest and best known effects of the syphilitic poison, leading to hemorrhagic discharges from the mouth, the bowels, the bladder, or the nose; to blood accumulations under the skin and mocosæ, or in the serous cavities and internal organs; or finally, making the syphilitic eruption itself hemorrhagic. The author emphasizes the importance of remembering these facts in the treatment of infants who have hemorrhagic discharges or a hemorrhagic eruption the cause of which is obscure.—*Archives of Pediatrics,* June, 1898.

